# Identification of Stemness-Related Genes for Cervical Squamous Cell Carcinoma and Endocervical Adenocarcinoma by Integrated Bioinformatics Analysis

**DOI:** 10.3389/fcell.2021.642724

**Published:** 2021-03-25

**Authors:** Hongjun Guo, Siqiao Wang, Min Ju, Penghui Yan, Wenhuizi Sun, Zhenyu Li, Siyu Wu, Ruoyi Lin, Shuyuan Xian, Daoke Yang, Jun Wang, Zongqiang Huang

**Affiliations:** ^1^Department of Orthopedics, The First Affiliated Hospital of Zhengzhou University, Zhengzhou, China; ^2^Department of Gynaecology, The First Affiliated Hospital of Zhengzhou University, Zhengzhou, China; ^3^Tongji University School of Medicine, Shanghai, China; ^4^Department of Radiotherapy, The First Affiliated Hospital of Zhengzhou University, Zhengzhou, China; ^5^Department of Pediatric Rehabilitation, Third Affiliated Hospital of Zhengzhou University, Zhengzhou, China

**Keywords:** cervical cancer, cancer stemness, metastasis, prognosis, naringenin, epithelial mesenchymal transition

## Abstract

**Background:**

Invasion and metastasis of cervical cancer are the main factors affecting the prognosis of patients with cervical squamous cell carcinoma (CESC). Therefore, it is of vital importance to find novel biomarkers that are associated with CESC invasion and metastasis, which will aid in the amelioration of individualized therapeutic methods for advanced patients.

**Methods:**

The gene expression profiles of 10 metastatic and 116 non-metastatic samples were downloaded from The Cancer Genome Atlas (TCGA), where differentially expressed genes (DEGs) were defined. Weighted gene correlation network analysis (WGCNA) was employed to identify the stemness-related genes (SRGs). Univariate and multivariate regression analyses were used to identify the most significant prognostic key genes. Differential expression analysis of transcription factor (TF) and Gene Set Variation Analysis (GSVA) were utilized to explore the potential upstream regulation of TFs and downstream signaling pathways, respectively. Co-expression analysis was performed among significantly enriched TFs, key SRGs, and signaling pathways to construct a metastasis-specific regulation network in CESC. Connectivity Map (CMap) analysis was performed to identify bioactive small molecules which might be potential inhibitors for the network. Additionally, direct regulatory patterns of key genes were validated by ChIP-seq and ATAC-seq data.

**Results:**

DEGs in yellow module acquired *via* WGCNA were defined as key genes which were most significantly related to mRNAsi. A multivariate Cox regression model was constructed and then utilized to explore the prognostic value of key SRGs by risk score. Area under curve (AUC) of the receiver operating characteristic (ROC) curve was 0.842. There was an obvious co expression pattern between the TF *NR5A2* and the key gene *VIM* (*R* = 0.843, *p* < 0.001), while *VIM* was also significantly co-expressed with hallmark epithelial mesenchymal transition (EMT) signaling pathway (*R* = 0.318, *p* < 0.001). Naringenin was selected as the potential bioactive small molecule inhibitor for metastatic CESC based on CMap analysis.

**Conclusions:**

*VIM* positively regulated by *NR5A2* affected EMT signaling pathways in metastatic CESC, and naringenin was the inhibitor for the treatment of metastatic CESC *via* suppressing cancer stemness. This hypothetical signaling axis and potential inhibitors provide biomarkers and novel therapeutic targets for metastatic CESC.

## Introduction

Cervical cancer refers to the second most prevalent gynecological cancer (Barth, 2020), being a major cause of women mortality (Small et al., 2017). As per the World Health Organization (WHO), about 530,000 women worldwide develop cervical cancer, and more than 270,000 women die from it every year (Lahue et al., 2015). Among all cervical cancers, the cervical squamous cell carcinoma (CESC) and endocervical adenocarcinoma (CESC) account for approximately 15% of female tumor deaths, presenting the second-highest mortality (Ojesina et al., 2014). Squamous cell carcinomas are most likely to arise from the ectocervix, accounting for about 75% of the invasive cervical carcinoma cases, while tumors arising from the endocervix are most likely to be adenocarcinomas. Tumor invasion and metastasis are the major factors affecting the prognosis of patients diagnosed with cervical cancers (Long, 2007; Kisseljov et al., 2008). Under most circumstances, patients have already progressed into moderate and advanced stages when diagnosed. Primary therapeutic methods for patients with cervical cancers include surgery or a concurrent chemoradiotherapy regimen that consists of cisplatin chemotherapy with brachytherapy and external beam radiotherapy (Small et al., 2017). Satisfactory results have been achieved with clinical trials including the human papillomavirus (HPV) vaccines, adoptive T-cell therapy, and checkpoint inhibitors (Paovonen et al., 2009; Small et al., 2017). With state of the art treatment, the 3 years local control rate of early-stage CESC patients is 87–95%. However, once patients develop local invasion and distant metastasis, the survival rate of which is significantly reduced, accompanied by increasing complications and the loss of radiotherapy opportunities. Hence, it is important to find novel biomarkers that are associated with CESC invasion and metastasis, which will aid in the amelioration of individualized therapeutic methods for advanced patients.

Cancer stemness cells (CSCs) are appealing targets for cancer therapy due to their self-renewal and multi-lineage differentiation abilities, which drive tumor growth and heterogeneity (Ayob and Ramasamy, 2018). CSCs exhibit more aggressive behaviors than normal cancer cells, thereby promoting tumor invasion and metastasis (Lopez et al., 2012;

Afify and Seno, 2019). It has been recently reported that the recurrence and radio/chemotherapy resistance of cervical cancer are owing to the presence of CSCs (Kim et al., 2012b; Ayob and Ramasamy, 2018). CSCs lead to genetic heterogeneity in cervical cancers, thereby reducing the effects of conventional anticancer therapies and facilitating the process of tumor invasion and metastasis (Cooke et al., 2011; Ortiz-Sanchez et al., 2016). In this clinical context, targeting CSCs can contribute to a better therapeutic outcome for CESC, whereas the research is not enough on this topic (Sato et al., 2016; Choi et al., 2021). Therefore, this study is innovative in stemness-related CESC biomarkers and individualized therapeutic methods. The prognosis of CESC was expected to be improved if the stemness-related signaling axes were found and affected by molecular targeted drugs.

Using stemness indices, an algorithm (Flemming, 2015; Malta et al., 2018) was designed to evaluate the similarity between cancer cells and stem cells. At the same time, mRNAsi is an index computed according to the molecular profiles of cell types with different degrees of stemness. Higher mRNAsi score is correlated well with activated biological processes in CSCs and stronger dedifferentiation capability, as reflected by clinical stages and histopathological grades. Hence, mRNAsi aids us in understanding the purity of tumor. RNA sequencing (RNA-seq) data and clinical information of CESC samples were acquired from The Cancer Genome Atlas (TCGA) database (Hutter and Zenklusen, 2018) to calculate the mRNAsi score.

Weighted gene correlation network analysis (WGCNA) is a systems biology method to identify gene association patterns. Based on mRNAsi and WGCNA, genes that had the strongest CESC-related correlation were screened out. Then, an independent prognostic model and a stemness-related gene (SRG) regulatory network for CESC were constructed. Importantly, transcription factors (TFs) binding information were crucial for understanding how genes were regulated, therefore Chromatin immunoprecipitation sequencing (ChIP-seq) analysis was performed to determine the direct regulatory pattern between TFs and key genes. Its related mechanisms were explored using the Assay for Transposase-Accessible Chromatin with high-throughput sequencing (ATAC-seq) analyses.

## Materials and Methods

### Data Collection

RNA-seq expression profiles of 126 CESC samples were obtained from TCGA database^1^. Gene names were replaced from Ensemble IDs to gene symbols using the Ensemble database^2^. We also extracted demographic information (age, gender, ethnicity, and so on), survival endpoint (days to death, vital status, and days to last follow-up), clinical stage, and histological type of these samples. In addition, samples with incomplete clinical information were excluded.

### Acquisition of the mRNAsi

Based on the normalized gene expression profiles, mRNA stemness index (mRNAsi) of each sample was computed using one-class logistic regression machine learning (OCLR) machine-learning algorithm (Malta et al., 2018). The mRNAsi was represented using an index between zero to one to indicate that the higher the mRNAsi, the greater the tumor dedifferentiation and higher activity of cancer stem cells.

### Differentially Expressed Genes Identification and Functional Annotation

The “edgeR” package was utilized to identify differentially expressed genes (DEGs) between metastasis and non-metastasis samples (Robinson et al., 2010). The absolute value of log2 Fold Change (FC) > 1.0 and False Discovery Rate (FDR) value < 0.05 were the screening criteria for DEG selection. Moreover, heat maps and volcano plots of DEGs were constructed using the limma and heat map packages (Ritchie et al., 2015). In addition, Gene Oncology (GO) and Kyoto Encyclopedia of Genes and Genomes (KEGG) functional enrichment analyses were performed to explore the signaling pathways where DEGs were enriched using the “cluster Profiler” R package with thresholds of *p* < 0.01 and FDR < 0.05 (Yu et al., 2012).

### Quantification of Hallmarks of Cancer Gene Sets by GSVA

In total, 50 hallmarks of cancer pathways were obtained from the Molecular Signatures Database (MSigDB) v7.0^3^ (Liberzon et al., 2015). The absolute quantification of these hallmark pathways was evaluated *via* Gene set variation analysis (GSVA) to select differentially expressed pathways between non-metastatic and metastatic samples.

### WGCNA

#### Construction of the Co-expression Network

A co-expression network demonstrating the correlations among DEGs, mRNAsi, and hallmarks of cancers was constructed with the WGCNA R package (Langfelder and Horvath, 2008). Initially, we removed genes with large deletions and outliers by filtering RNA-seq data. Next, we performed a co-expression analysis of pair-wise genes using Pearson correlation coefficients. Subsequently, as described earlier, the weighted adjacency matrix was constructed based on the power function: a_ij_ = | s_ij_| ^β^ (s_ij_ = Pearson correlation between gene *i* and gene *j*; a_mn_ = the weighted network adjacency between gene *i* and gene *j*; β ≥ 1). We selected a suitable value of β to enhance the similarity of the matrix. Moreover, in order to better investigate gene connectivity in this network, we converted the adjacency matrix to a topological overlap matrix (TOM). Eventually, an average linkage hierarchical clustering with TOM-based heterogeneity was performed to build module dendrograms.

### Screening of Key Genes in Modules

We deliberated the relationship between the modules and hallmarks of cancer as well as mRNAsi score in which mRNAsi and hallmarks of cancer were used as clinical phenotypes for further analysis. Gene significance (GS) was computed to evaluate the correlation between sample traits and genes. Likewise, the correlations between modules and sample traits were defined as module significance (MS) *via* computing the average absolute GS of genes from the relevant modules. Additionally, module eigengenes (MEs) were the central components of module genes, which were representatives of the gene expression profiles. To assess the correlation degree of each gene and ME, Module Membership (MM) was computed to evaluate the correlativity between gene expression profiles and MEs. Pearson’s correlation analysis was performed for evaluation of the correlations among MEs, MM, and sample traits.

To identify SRGs, mRNAsi was primarily considered as the phenotype of interest. Pre-determining cutoffs standards were defined as cor. gene GS > 0.3 and cor. gene MM > 0.3. Hence, module with a comparatively high MS was suggested as the key module and was preserved for identification of SRGs ulteriorly. Additionally, hallmark pathways which were noteworthily correlated with the key module were considered as biological processes or putative pathways, mediating SRGs to function. Hallmark pathways with *p* value < 0.05 and correlation coefficient with the key module > 0.05 were statistically significant. Furthermore, the selected Hallmark pathways were preserved for further analysis.

### Identification of Prognostic Key Genes and Construction of Prognostic Model

Univariate Cox analysis was conducted to screen significant key genes, which were incorporated into an initial multivariate Cox regression model. Further, the Least Absolute Shrinkage and Selection Operator (LASSO) regression was utilized to filter the independent variables with great significance and reduce over-fitting phenomenon (Subramanian et al., 2017). Moreover, the variables were incorporated into the final multivariate Cox regression model. In addition, accuracy of this model was assessed by receiver operator characteristic (ROC) curve. Further, for each CESC sample, the risk score was computed on the gene expression level, and the formula (Uhlen et al., 2015) is as follows:

Risk⁢score=β⁢1×gene⁢ 1+β⁢2×gene⁢ 2+β⁢3×gene⁢ 3⁢…⁢…+β⁢n×gene⁢n

Specifically, the order number of relevant gene in the model was defined as “*n*,” regression coefficient of a gene was defined as “β,” and gene_n_ indicated expression level of the *n*th key gene for each sample, correspondingly.

Moreover, according to the median risk score, samples were categorized into low and high risk group. Kaplan–Meier curve was used to test the prognosis value of the multivariate model. Then, the risk curve and scatter plot were constructed to reorder these samples. In addition, multivariate Cox analysis was rectified by demographics and tumor information to evaluate the prognostic value of the risk score in relation to age, TNM stage, and clinical stage.

### Identification of the Upstream TFs

318 cancer related TFs were retrieved from the Cistrome database^4^ (Zheng et al., 2019). Further, we conducted co-expression analysis to identify the upstream TFs which were significantly correlated with the key genes. TFs with correlation coefficients greater than 0.50 were extracted for subsequent analysis.

### Identification of the Downstream Signaling Pathways

As discussed above, GSVA was performed to quantify the 50 hallmarks of cancer gene sets in each sample. In addition, Gene Set Enrichment Analysis (GSEA) was also employed to calculate Fragments Per Kilobase per Million (FPKM) from the raw RNA-seq data with 50 hallmarks of cancer. In order to identify the hallmarks of cancer that were significantly correlated with the module we focused on as potential downstream pathways, differential expression analysis was utilized. Specifically, significant hallmarks of cancer differently expressed between metastasis and non-metastasis were extracted for further analysis, where FDR value < 0.05 was set as a screening criterion. Moreover, the intersection of the hallmarks of cancer acquired *via* significantly differential expression analysis in GSVA and WGCNA were eventually defined as the potential downstream signaling pathways for subsequent analysis.

### Regulatory Network of TFs, Key Genes and Hallmarks of Cancer

Co-expression Pearson correlation analysis was conducted based on TFs, SRGs, and Hallmark gene sets. And a regulatory network of the three components mentioned above was constructed using Cytoscape (3.7.1). Interaction pairs between SRGs and TFs and hallmark gene sets were controlled for | correlation coefficient| > 0.40, *p* value < 0.05 and | correlation coefficient| > 0.30, *p* value < 0.05, respectively.

### Connectivity Map Analysis

Connectivity Map (build 02) (CMap) was used to explore small-molecule compounds which may target cancer SRGs. The CMap^5^ gather up genomic signatures for researchers to identify potential compounds for tumor therapeutics (Lamb et al., 2006; Subramanian et al., 2017).

The mechanism of actions (MoA)^6^ of target compounds were securable in the CMap database, including compounds (perturbation) information, such as transcriptional responses of human cells to perturbagens, protein target, and structural formula. Based on the MoA, compounds which may target TFs, SRGs, and Hallmark gene sets in this study were extracted for further validation.

### Validation of the Regulatory Mechanism of Transcription Factors

We performed two algorithms [JASPAR (Khan et al., 2018) and ENCODE (2011) TF Targets] to re-predict the transcriptional regulation pattern between key upstream TFs and SRGs to further undergird our hypothesis. ChIP-seq data in the Cistrome database^7^ (Zheng et al., 2019) were used to validate the transcriptional regulatory relationships of the pair-wise genes mentioned above.

### ATAC-seq Validation

Initially, we downloaded the ATAC-seq data of CESC samples from the TGCA project of chromatin accessibility landscape of primary human cancers^8^ and identified the chromatin accessibility in the location of these biomarker genes (Corces et al., 2018). Furthermore, we further verified the binding relationship *via* comparing with control groups, which was achieved using the Gviz package and based on the original ATAC-seq data and ChIP-seq data in Cistrome database (Hahne and Ivanek, 2016; Li et al., 2019).

### External Validation and Gene Sets Over Representation Analysis

Gene expression profiles of 19 cervical cancer patients with positive lymph nodes (N+) and 20 patients with negative (N0) were downloaded from GSE26511 (Gene Expression Omnibus, GEO)^9^ (Noordhuis et al., 2011). DEGs between metastasis and non-metastasis samples were identified using the “edgeR” package (Robinson et al., 2010). The absolute value of log2 Fold Change (FC) > 1.0 and False Discovery Rate (FDR) value < 0.05 were the screening criteria for DEG selection. In addition, a volcano plot of DEGs was constructed using the limma packages (Ritchie et al., 2015).

Gene sets over-representation analysis (GSORA), a common technique of enrichment analysis, evaluates the fraction of interested genes (e.g., DEGs) which belong to tested clusters (e.g., hallmark signaling pathways). In the present study, 49 CESC-related hallmark signaling pathways obtained from the MSigDB were categorized into nine clusters based on similar functional characteristics, including cluster C1 to 8 and H (Liberzon et al., 2015). Further, GSORA was performed to identify the functional enrichment of DEGs in the MSigDB gene sets.^10^^,^^11^

### Statistics Analysis

In this study, the R software (Institute for Statistics and Mathematics, Vienna, Austria;^12^ version 3.6.1) was applied for all statistics analysis processes. It was statistically significant only when two-sided *p* value < 0.05 (Package: e1071, parallel, preprocessCore, sva, limma, edgeR, ggplot2, survminer, survival, rms, randomForest, pROC, glmnet, pheatmap, timeROC, vioplot, corrplot, ConsensusClusterPlus, forestplot, survivalROC, beeswarm, edgeR, chromVAR, Biostrings, BSgenome.Hsapiens.UCSC.hg38, ChIPseeker, TxDb.Hsapiens.UCSC.hg38.knownGene, clusterProfiler, org.Hs.eg.db, ggplot2, karyoploteR, GSVA, GSEABase, stringr, GEOquery, dplyr, ComplexHeatmap, RColorBrewer, tibble, cowplot, ggcorrplot, xlsx, tidyverse, GEOquery, plyr, and circlize).

## Results

### DEGs Identification and Functional Annotation

**FIGURE 1:**
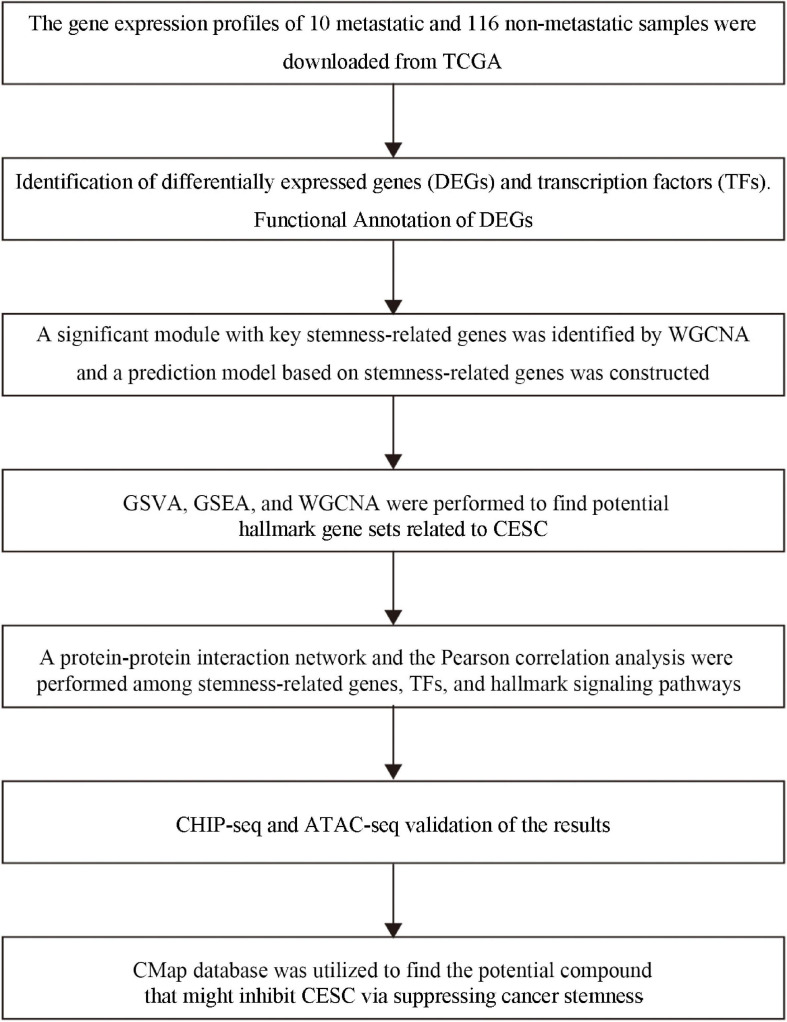
The flow chart of the analysis process. TCGA, the Cancer Genome Atlas; MsigDB, Molecular Signatures Database; KEGG, Kyoto Encyclopedia of Genes and Genomes; WGCNA, Weighted Gene Correlation Network Analysis; GSVA, Gene Set Variation Analysis; GSEA, Gene-Set Enrichment Analysis.

**FIGURE 2:**
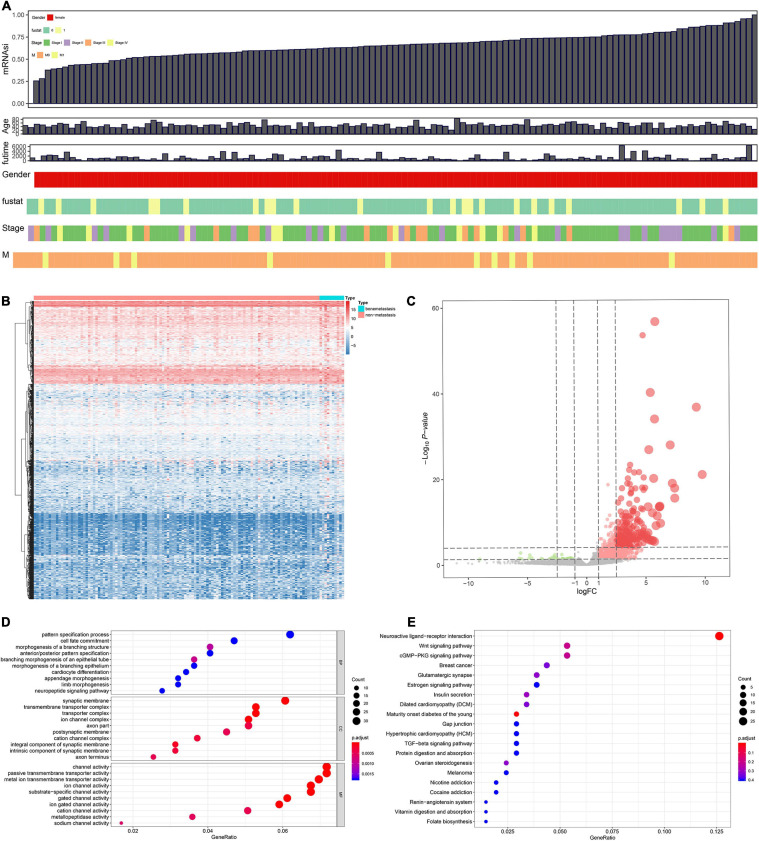
Combinative analyses based on gene expression stemness indices **(A)**. Heat map for the differentially expressed genes (DEGs) between 10 metastatic and 116 non-metastatic patients with the cervical squamous cell carcinoma and endocervical adenocarcinoma (CESC) **(B)**. Volcano plot for DEGs between 10 metastatic and 116 non-metastatic patients with CESC **(C)**. The functional enrichment analysis for these DEGs in Gene Ontology (GO) terms **(D)** and Kyoto Encyclopedia of Genes and Genomes (KEGG) pathways **(E)**.

The analysis procedure of this study was summarized in Figure 1. Combinative analysis based on gene expression stemness indices was performed by using the OCLR machine learning algorithm (Figure 2A).

Differentially expressed genes s identified between 10 metastasis and 116 non-metastasis CESC samples were demonstrated by the heat map plot (Figure 2B) and volcano plot (Figure 2C).

Gene oncology and KEGG analyses were used to annotate the function of DEGs. The results showed the most significant GO terms for BP, CC, and MF were pattern specification process, synaptic membrane, and channel activity (Figure 2D). In addition, KEGG analysis showed that the functional similarities mainly enriched in neuroactive ligand–receptor interaction (Figure 2E).

### WGCNA

**FIGURE 3:**
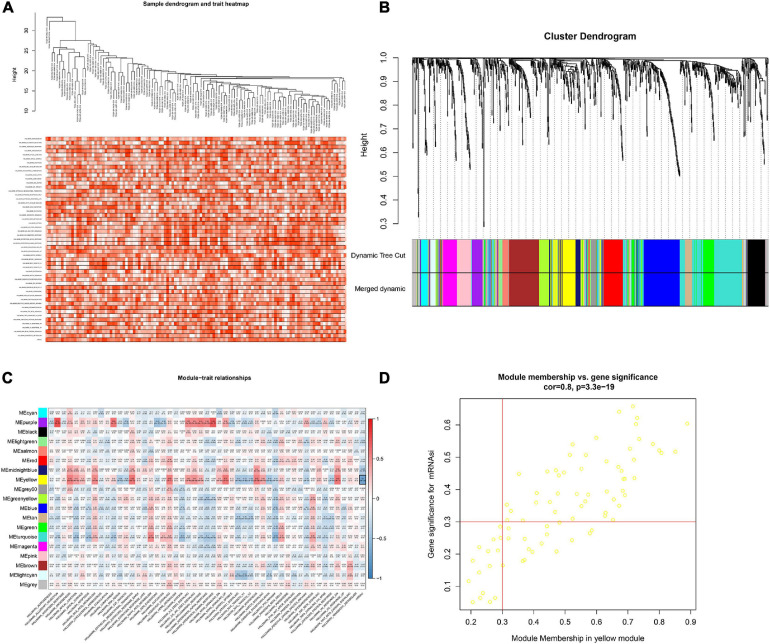
Clustering based on the transcriptional level of 50 hallmark gene sets in chordoma samples **(A)**. Hierarchical clustering tree developed by the weighted correlation coefficients. Each branch represents a co-expression module in different colors **(B)**. Heatmap showing the correlation between modules and hallmark gene sets. The framed yellow module was the key module which was most relevant to mRNAsi. Gene Significance (GS) and its corresponding *p* value were computed and shown in the heatmap **(C)**. Scatter diagram showing the correlation between gene significance for hallmarks of cancer and Module Membership in yellow module **(D)**.

**FIGURE 4:**
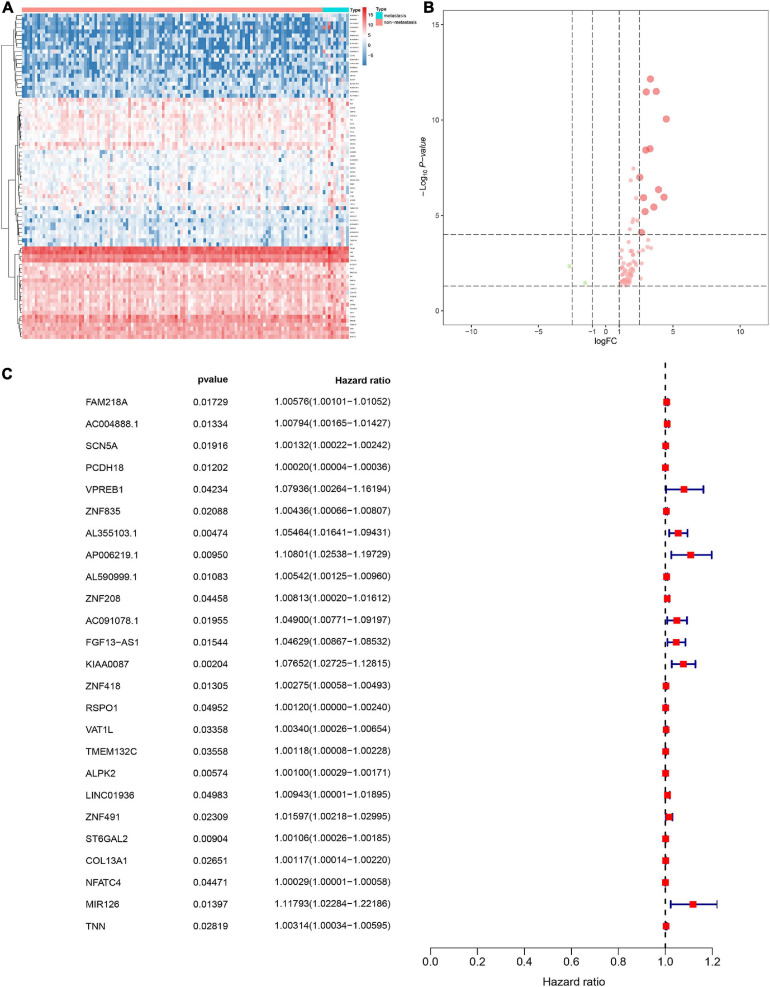
Heat map for the differentially expressed stemness-related genes (DESRGs) between 10 metastatic and 116 non-metastatic patients with CESC **(A)**. Volcano plot for DESRGs between 10 metastatic and 116 non-metastatic patients with CESC **(B)**. The proportional hazards model based on 25 key DEMRGs **(C).**

In order to screen genes that were significantly correlated with CESC, a DEGs co-expression network was constructed to select stemness indices-related modules based on the TCGA datasets using WGCNA. In this study, the soft threshold β = 4 was adopted to achieve the scale-free topology criterion of the network (Figures 3A,B). We extracted 19 gene modules in different colors with genes which have similar expression patterns for further analysis (Figure 3C). To analyze the correlation between the gene modules and mRNAsi score, MM was defined as the overall gene expression level of the relevant module to calculate the correlations with phenotypes. Meanwhile, the correlation between gene expression and hallmarks of cancer was investigated. Importantly, the yellow module showed the highest negative correlation with mRNAsi score with a correlation close to −0.7. Thus, we considered the yellow module as the key module, from which key genes were extracted with the selection criteria of cor.MM > 0.3 and cor.GS > 0.3 (Figure 3D). Besides, differential expression analysis was also utilized to screen differentially expressed stemness-related genes (DESRGs) with | log2 FC| > 1.0 and FDR value < 0.05 based on the key genes aforementioned (Figures 4A,B). Eventually, 81 DESRGs were extracted for subsequent analysis. Meanwhile, 18 hallmarks of cancer significantly correlated with yellow module were also identified for further analysis.

### Identification of the Prognostic Model

**FIGURE 5:**
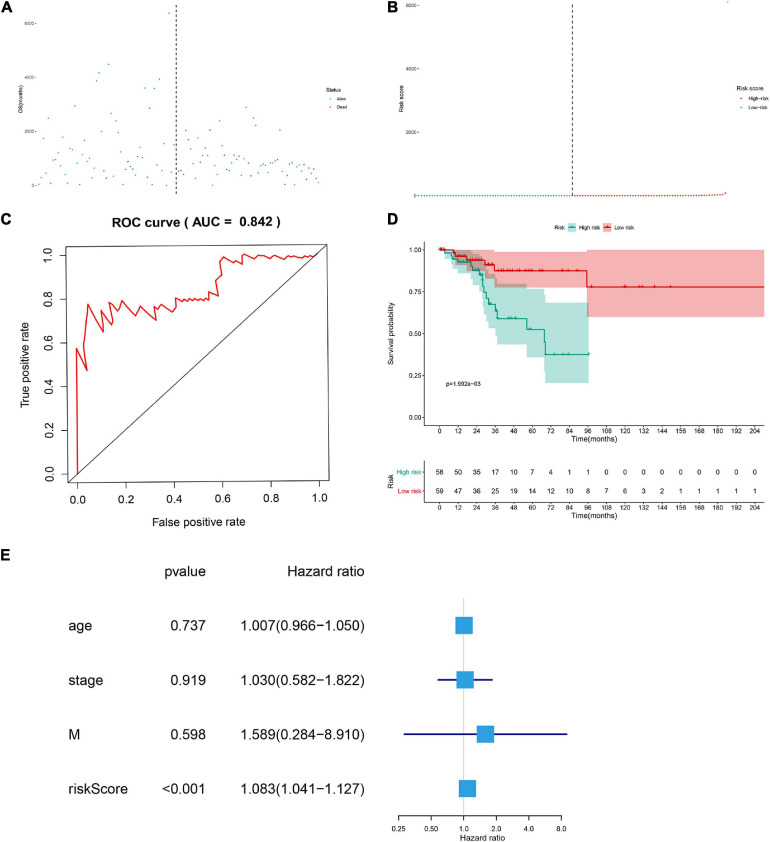
The scatter plot of the samples **(A)**, the risk curve of each sample reordered by risk score **(B)**, green and red represent low risk and high risk groups, respectively. ROC curve (AUC = 0.921) for prognostic DESRGs **(C)**. Overall survival Kaplan–Meier curve for prognostic DEMRGs (*p* < 0.001) **(D)**. Univariate Cox regression models indicated the risk score was an independent prognostic factor **(E)**.

The DESRGs were integrated into a proportional hazards model to evaluate the prognosis value in CESC patients (Figure 4C). Furthermore, these genes were incorporated in a multivariate Cox model, the formula of which was utilized to compute the risk score for each CESC patient. CESC samples were divided into low-risk and high-risk groups based on the median of risk score. Scatter plot and risk curve illustrated the risk score and vital status among patients with CESC (Figures 5A,B). The area under receiver operating characteristic (ROC) curve was calculated to quantify the predictive accuracy of the model (Figure 5C), which was of satisfactory prognostic value (area under curve, AUC = 0.842). In addition, the Kaplan–Meier survival curve showed the survival rate in the low-risk group was significantly prolonged than those in high-risk group, suggesting the great prognostic value of risk score (Figure 5D). Moreover, in multivariate Cox regression analysis, the risk score represented an independent prognostic indicator [HR = 1.083, 95% CI (1.041–1.127), *p* < 0.001] (Figure 5E).

### Identification of Upstream TFs, Key Genes, and Downstream Signaling Pathways

**FIGURE 6:**
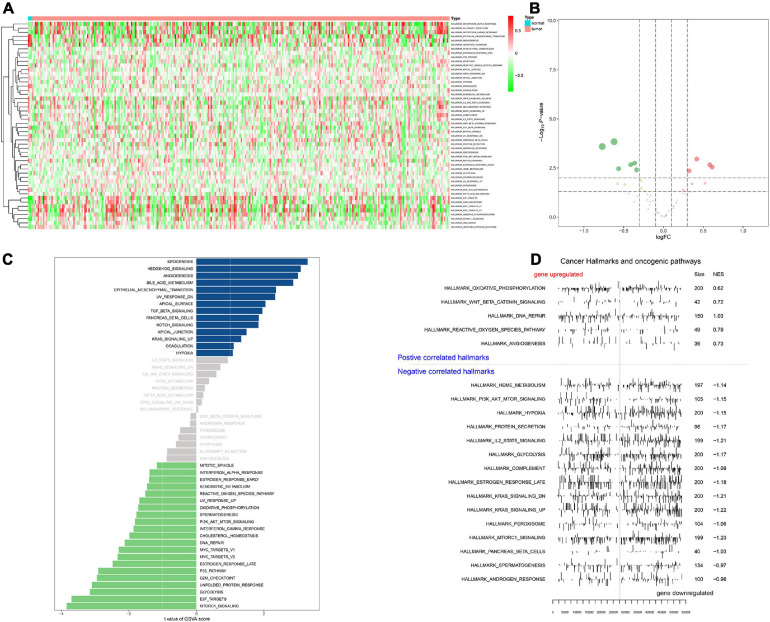
Heat map for Gene set variation analysis (GSVA) showing the co-expression level of 50 hallmark gene sets in CESC samples **(A)**. Volcano plot for hallmark signaling pathways, green and red dots represented significant differential expression in PBMC samples **(B)**. Bar plot revealing the *t* value of GSVA score **(C)**. Positive correlated hallmarks and Negative correlated hallmarks acquired by GSEA **(D)**.

**FIGURE 7:**
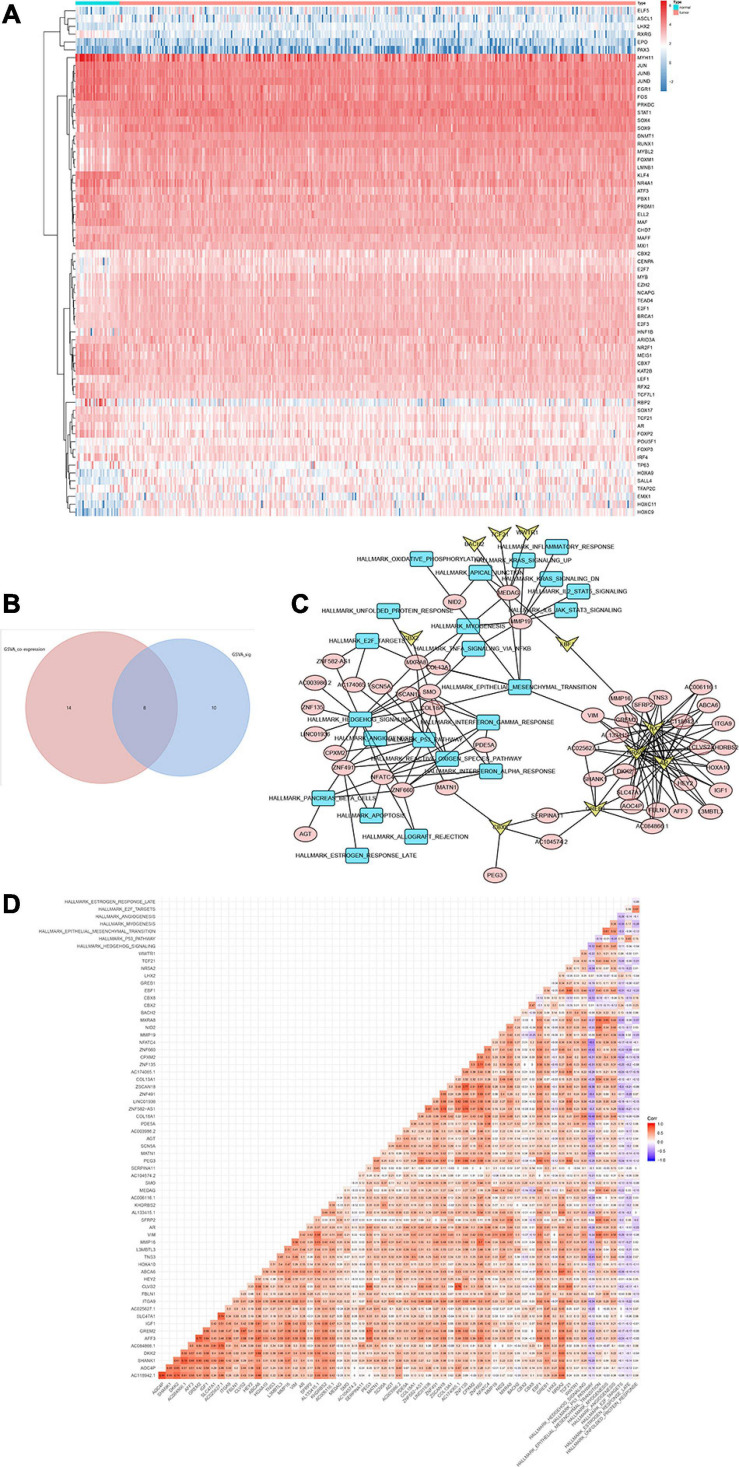
Heat map showing the expression level of 65 differential expressed transcriptional factors (TFs) between 10 metastatic and 116 non-metastatic patients with CESC **(A)**. Venn plot for hallmarks of cancer *via* GSVA. Eight downstream mechanism were extracted from the intersection **(B)**. Regulatory network of TFs, DESRGs, and hallmark signaling pathways. Arrows represented TFs. Ellipses represented DESRGs. Rectangles represented hallmark signaling pathways **(C)**. Heat map for the correlation analysis (Pearson analysis) of DESRGs, TFs, and hallmark signaling pathways **(D)**.

Heat map (Figure 6A) and volcano map (Figure 6B) illustrated the differential expression levels of 50 hallmarks of cancer between CESC and normal tissues. Besides, the differential expression levels of 50 hallmarks of cancer were further assessed by GSVA and GSEA, respectively (Figures 6C,D). Meanwhile, EdgeR method was utilized to identify differential expressed TFs with FDR value < 0.05. Heat map illustrated 65 differentially expressed TFs extracted from 318 TFs (Figure 7A). Co-expression analysis was conducted to identify the upstream TFs which were significantly correlated with the key genes. TFs and key genes with | correlation coefficient| > 0.50 and *p* value < 0.05 were extracted for subsequent analysis. In total, based on 22 hallmarks of cancer that were significantly co-expressed and 18 hallmarks of cancer that were significantly differently expressed between metastasis and non-metastasis samples *via* GSVA, eight downstream mechanism were extracted from the intersection (Figure 7B). Moreover, co-expression analysis was conducted among TFs, DESRGs, and hallmarks of cancer, the co-expression interaction pairs were utilized to construct the regulatory network (Figure 7C). Subsequently, to quantify the interaction coefficients among 22 Hallmark gene sets, 10 TFs, and 48 DESRGs, co-expression analyses were conducted at the transcriptional level (Figure 7D). There was an obvious co expression pattern between the TF *NR5A2* and the key gene *VIM* (*R* = 0.843, *p* < 0.001), and *VIM* was significantly co-expressed with hallmark epithelial mesenchymal transition (EMT) pathway (*R* = 0.318, *p* < 0.001). Eventually, we put forward a scientific hypothesis: *VIM* was positively regulated by *NR5A2* and hallmark EMT was the potential downstream pathway of *VIM* in CESC metastasis.

### Identification of Bioactive Small Molecules Inhibitor

**FIGURE 8:**
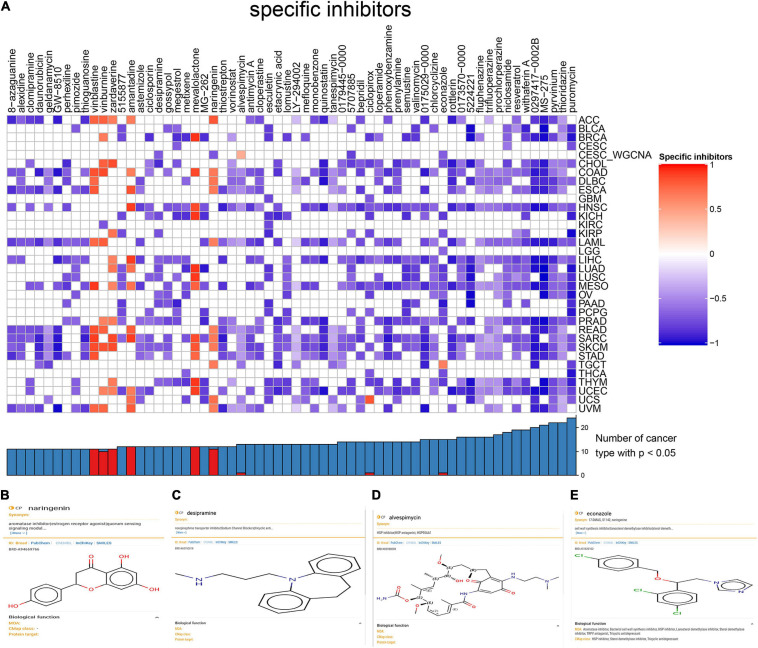
Heat map for small-molecule compounds from the CMap which might be capable of inhibiting CESC *via* suppressing cancer stemness **(A)**. Structural formulas and biological functions of naringenin **(B)**, desipramine **(C)**, alvespimycin **(D)**, and econazole **(E)**.

Statistically significant results were shown in a complex heat map demonstrating bioactive small molecules in over 10 types of cancers (Figure 8A). The results revealed that naringenin (Figure 8B), desipramine (Figure 8C), alvespimycin (Figure 8D), and econazole (Figure 8E) (*p* < 0.05) were the best compounds inhibiting CESC. After comprehensive analysis, naringenin was selected as bioactive small molecule inhibitor in CESC development by targeting DESRGs, and genes which were involved in hallmark EMT gene sets including *SNAL2* and *MMP2*.

### ChIP-seq Validation

**FIGURE 9:**
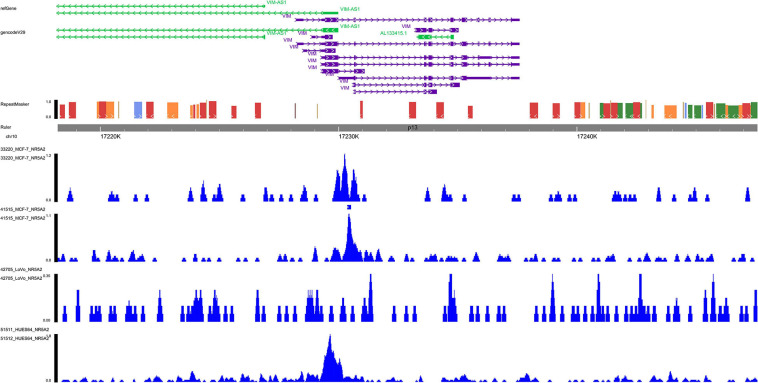
ChIP-seq data validation. In *NR5A2* ChIP-seq data, multiple binding peaks were found in *VIM* sequences. ChIP-seq, Chromatin immunoprecipitation sequence.

Based on ChIP-seq data of *NR5A2* in Cistrome database (homo sapiens), multiple binding peaks were found in *VIM* sequences (Figure 9). Therefore, we could determine the direct transcriptional regulatory relationship between *NR5A2* and *VIM*.

### ATAC-seq Validation

**FIGURE 10:**
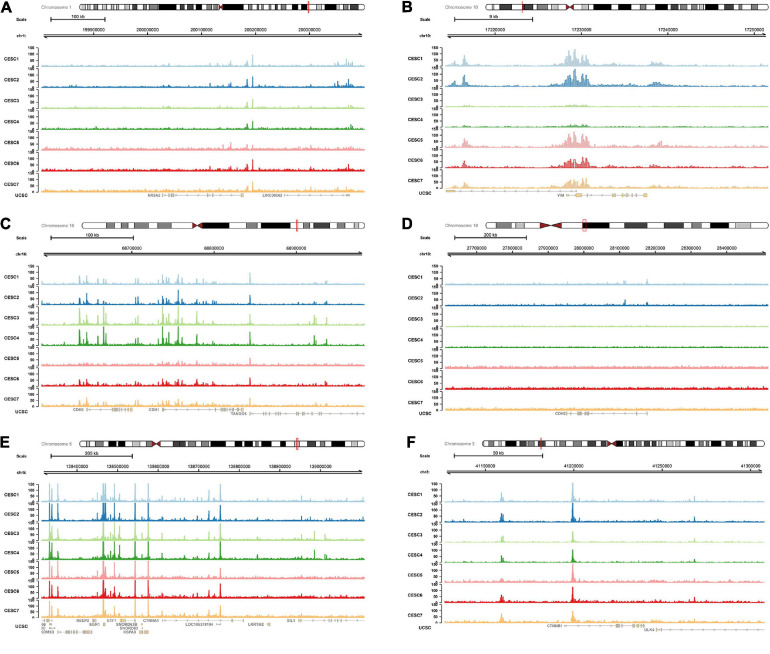
ATAC-seq data validation. In ATAC-seq data of CESC, multiple peaks were identified in *NR5A2* **(A)**, *VIM* **(B)**, *CDH1* **(C)**, *CDH2* **(D)**, *CTNNA1* **(E)**, and *CTNNB1* **(F)** sequences. ATAC-seq, assay for transposase-accessible Chromatin with high-throughput sequencing.

Multiple open chromatin regions in sorted CESC cells were identified using ATAC-seq analysis (Figure 10). There were strong ATAC-seq binding peaks in CESC cells at the *NR5A2* promoter and at known enhancers in the introns and in introns of neighboring genes, indicating these regions may function as enhancers of *NR5A2*.

**FIGURE 11:**
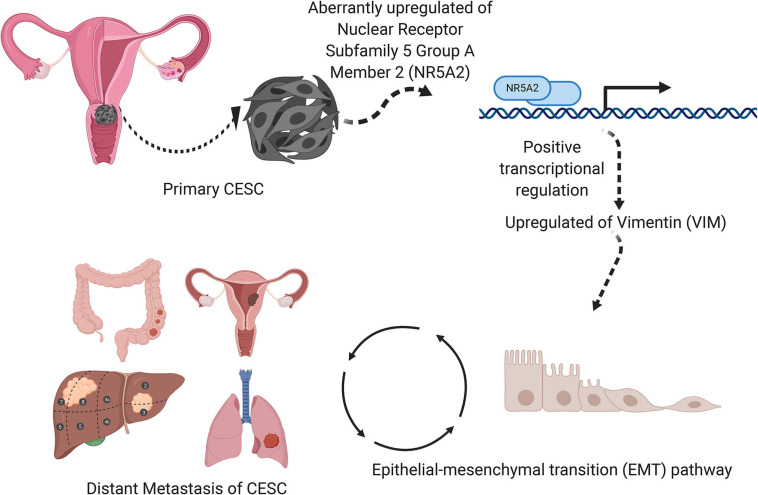
A speculatively schematic diagram of the scientific hypothesis including the most significant DESRG (VIM), TF (NR5A2), and downstream pathway (Hallmark epithelial mesenchymal transition).

A schematic diagram describing the mechanism of VIM, NR5A2, and hallmark EMT pathway in the metastasis and invasion of CESC was illustrated in Figure 11.

### External Validation and Gene Sets Over Representation Analysis

*NR5A2* (TF), *VIM* (key stemness-related gene), *MMP2* (EMT pathway gene), and *MMP9* (EMT pathway gene) were significantly up-regulated in metastasis CESC samples (Supplementary Figure 1A).

In the cluster H, DEGs mostly enriched in hallmark EMT pathway, which was consistent with the previous results (Supplementary Figure 1B).

## Discussion

Cervical cancer ranks the second most common gynecological malignant tumor and the fourth leading cause of female cancer death worldwide (Flanagan, 2018). Patients have already progressed into advanced stages when diagnosed in most cases. The standard therapeutic regimes for cervical cancer patients are concurrent chemoradiotherapy plus brachytherapy, whereas the prognosis is poor (Naga et al., 2018). Hence, potential biomarkers are urgently required to assess the risk of metastatic cervical cancer patients, as well as corresponding targeted drugs that can improve clinical outcomes.

In this study, a total of 126 samples, 318 TFs, and 50 hallmarks of cancer were obtained based on comprehensive bioinformation. DEGs between 10 metastasis and 116 non-metastasis samples were identified using edgeR. Further, GO and KEGG analyses were utilized for function annotation of DEGs. The yellow module was finally identified as our interest module *via* performing WGCNA, which is remarkably correlated with tumor stemness based on mRNAsi score. 81 key genes significantly related to cancer stemness were extracted using differential expression analysis. Additionally, univariate Cox regression and multivariate Cox regression were applied to assess the prognostic value of key genes and risk score, respectively. *VIM* was speculated as the most significant prognosis gene. A prognostic model based on aforesaid 81 key genes was developed and possessed a significantly high reliability (Kaplan–Meier curve *p* value < 0.001, AUC = 0.894), which could effectively predict the survival outcomes of CESC patients, suggesting a positive interaction between mRNAsi and poor prognosis. Furthermore, based on significant correlation analysis between TFs and key genes, the *NR5A2* (TF) and *VIM* (key gene) pair was considered as significant (*R* = 0.84305, *p* < 0.001, positive). Based on correlation analysis between *VIM* and 50 hallmarks of cancer, hallmark EMT (*R* = 0.318073, *p* < 0.001, positive) was identified as the most significant downstream pathway. Ultimately, we postulated *VIM* was positively regulated by *NR5A2* and the EMT was the potential downstream of *VIM* in CESC metastasis. In addition, naringenin was identified as the most important bioactive small molecule inhibitor suppressing CESC metastasis *via* targeting *NAL2* and *MMP2*, which were involved in hallmark EMT gene sets.

Chromatin immunoprecipitation (ChIP) allows researchers to assess the recruitment of a specific protein at a given locus with a high resolution, on average in the cell population. When combined with high-throughput ChIP-seq data, it can provide a special and convenient method to analyze at a genome-wide scale. The regulatory relationship between *NR5A2* and *VIM* was validated using the ChIP-seq analysis, and it showed a strong connection between them. ATAC-seq, a method map chromatin accessibility genome-wide, is an impressively flexible, simple, and powerful technique. ATAC-seq reads can be utilized for inferring regions of increased accessibility, as well as for mapping regions of nucleosome position and TF binding (Buenrostro et al., 2013). We correlated the maps of chromatin with RNA-seq data from CESC samples to identify the *cis*-regulatory elements which may be implicated in the regulation of *VIM* induced by *NR5A2*.

The orphan nuclear receptor *NR5A2*, a transcriptional factor, plays a critical role in steroidogenesis, normal differentiation, cholesterol transport, and bile-acid homeostasis (Lu et al., 2000; Fayard et al., 2004). Additionally, *NR5A2* is implicated in the maintenance of pluripotency in embryonic stem cells (ESCs) (Gu et al., 2005) and reprogramming of somatic cells into induced pluripotent stem cells (iPSCs) (Heng et al., 2010; Wang et al., 2011). Recently, accumulating evidence has also shown the participation of *NR5A2* in the pathogenesis of various tumors including cervical cancers. In this study, *NR5A2* was identified to express differentially between metastatic and non-metastatic tissues of cervical cancers, and may promote the tumorigenesis and metastasis by regulating *VIM*.

The *VIM* gene is located on chromosome 10p13, encoding a member belonging to a family of intermediate filaments that maintain cytoarchitecture and tissue integrity, which influences the regulation of multiple cellular functions (Fuchs and Weber, 1994). *VIM* is the primary intermediate filament protein in mesenchymal cells including fibroblasts (Osborn and Weber, 1984). *VIM* is implicated in maintaining cell shape and integrity of the cytoplasm, and stabilizing cytoskeletal interactions. It also operates as an organizer of other crucial proteins related to cell attachment, migration, and signaling (Danielsson et al., 2018; Yu et al., 2018). Particularly, *VIM* is considered as a biomarker of epithelial-mesenchymal transition, a cellular reprogramming process where epithelial cells acquire a mesenchymal phenotype which causes them to alter shape and to exhibit increased motility (Thiery, 2002). Thus, *VIM* methylation may be a novel prognostic marker for CESC. In this study, *VIM* was significantly correlated with mRNAsi based on WGCNA and therefore involved in the progression of CESC. Whereas, there was still no research on the direct connection between *NR5A2* and *VIM*, we speculated that *NR5A2* participated in *VIM* transcription based comprehensive bioinformation analysis.

Hallmark EMT is generally correlated with tumorigenesis, malignant progression, invasion, and metastasis, seriously affecting the life quality of patients. EMT is considered the first step for infiltration and metastasis of tumor cells. Besides, EMT is often defined by downregulated expression of epithelial biomarkers (such as *E*-cadherin) and increased expression of mesenchymal biomarkers (such as *N*-cadherin and *VIM*) (Pastushenko and Blanpain, 2019). Moreover, EMT-associated TFs, such as *ZEB* (*ZEB1* and *ZEB2*), *SNAl* (*SNAI1* and *SNAI2*), and *TWIST* (*TWIST1* and *TWIST2*) nuclear proteins, can suppress *E*-cadherin expression and regulate the EMT process *via* different pathways (De Craene and Berx, 2013). Further, a recent study demonstrates that activation of the phosphatidylinositol 3’ kinase (PI3K)/*AKT* axis is emerging as a central character of EMT (Larue and Bellacosa, 2005). EMT is a significant process implicated in cancer cell invasion and metastasis. Inhibiting EMT may suppress or even block the invasion and metastasis of cervical cancer, which may provide a new theoretical basis for the treatment of CESC.

To our knowledge, stemness is postulated to be a crucial role in CESC invasion and metastasis. Inhibiting genes associated with stemness and stimulating cell differentiation may assist in the selection of treatment strategies for CESC. Naringenin was considered as the most significant bioactive small molecule compound inhibiting CESC metastasis *via* suppressing expression of DESRGs based on CMap analysis. *SNAI2* and *MMP2*, genes involved in hallmark EMT, were potential targets for naringenin. Further, naringenin is able to inhibit cancer cell migration through the up-regulation of *E*-cadherin expression, but down-regulation of the expression of *VIM*, *SNAIL* family zinc finger 1(*SNAI1*), *SNAIL* family zinc finger 2(*SNAl2*) (Han et al., 2018). Moreover, it could also inhibit the *AKT* activities and induce the reduction of *MMP*-2 and -9 activities (Yang et al., 2008; Liao et al., 2014; Chang et al., 2017). The TGF-β signaling pathway serves as a critical regulator of EMT, which can trigger and regulate physiological functions of EMT. A recent study reports that naringenin significantly inhibits the transcription of *SMAD3* induced by TGF-β1 and reduces the probability of TGF-β1 binding to its receptor TβRII, thereby inhibiting receptor dimerization and downstream signaling transduction, inhibiting cell migration and invasion (Lou et al., 2012). Naringenin can also inhibit cell proliferation and induce cancer cell apoptosis through multiple mechanisms of estrogen receptor (ER), inducing reactive oxygen species (ROS) production, mitochondrial depolarization, and causing cell cycle arrest in G0/G1 phase. Moreover, naringenin has cytotoxic effect on cancer cell lines of the cervix (Hela, Hela-TG) (Kanno et al., 2005). It can also inhibit the proliferation of human squamous cell carcinoma and epidermoid carcinoma (Ahamad et al., 2014; Maggioni et al., 2014) and has anti-cancer effects on human cervical cancer cells (Kim et al., 2012a; Zaim et al., 2018). Hence, based on comprehensive bioinformatics and other studies, naringenin is considered as a promising drug which provides a novel therapeutic basis for patients with CESC.

Several inevitable limitations in this study should be taken into consideration. Firstly, data acquired from the public datasets were statistically incomplete. It’s far too difficult to reduce the potential error and bias *via* acquiring the same number of cases with different genders, age groups, and races, which may lead to the lack of comprehensiveness. Secondly, despite the results validated by external databases, the sample size was limited. Thirdly, the scientific hypothesis was mainly based on bioinformatics, and it was not validated by exploring the underlying molecular mechanisms. Therefore, ChIP-seq and ATAC-seq validation was performed to determine the direct transcriptional regulation pattern between the TFs and SRGs.

## Conclusion

In conclusion, *VIM* was regulated by *NR5A2*, and by effecting the EMT signaling pathway it was involved in CESC metastasis. In addition, naringenin was selected as inhibitor for cervical carcinoma metastasis by targeting the EMT. The hypothetical signaling axis in this study may provide candidate prognostic biomarkers and therapeutic targets for metastatic CESC.

## Data Availability Statement

Publicly available datasets were analyzed in this study. This data can be found here: The datasets generated and/or analyzed during the current study are available in the in the Supplementary Material, TCGA-CESC program (https://portal.gdc.cancer.gov), Cistrome database (http://cistrome.org/), and the TGCA project of chromatin accessibility landscape of primary human cancers (https://gdc.cancer.gov/about-data/publications/ATACseq-AWG).

## Ethics Statement

The studies involving human participants were reviewed and approved by the Ethics Committee of The First Affiliated Hospital of Zhengzhou University. The patients/participants provided their written informed consent to participate in this study. Written informed consent was obtained from the individual(s) for the publication of any potentially identifiable images or data included in this article.

## Author Contributions

All authors contributed to the conception/design, collection and/or assembly of data, data analysis and interpretation, and final approval of manuscript.

## Conflict of Interest

The authors declare that the research was conducted in the absence of any commercial or financial relationships that could be construed as a potential conflict of interest.

## Footnotes

1 https://tcga-data.nci.nih.gov

2 http://asia.ensembl.org/index.html

3 https://www.gsea-msigdb.org/gsea/msigdb/index.jsp

4 http://cistrome.org/

5 https://portals.broadinstitute.org/cmap/

6 http://clue.io/

7 http://cistrome.org/

8 https://gdc.cancer.gov/about-data/publications/ATACseq-AWG

9 https://www.ncbi.nlm.nih.gov/geo/query/acc.cgi?acc=GSE26511

10 https://github.com/tomastokar/gsoap

11 https://www.gsea-msigdb.org/gsea/msigdb/index.jsp

12 www.r-project.org

## Supplementary Material

Supplementary Figure 1 Volcano plot for DEGs between 19 cervical cancer patients with positive lymph nodes (N+) and 20 patients with negative (N0) **(A)**. Bar plot for gene sets over-representation analysis of DEGs and significant hallmark pathways **(B)**.
