# Supplementary figures and images for: Identification of Stemness-Related Genes for Cervical Squamous Cell Carcinoma and Endocervical Adenocarcinoma by Integrated Bioinformatics Analysis
Source: Front Cell Dev Biol. 2021 Mar 25;9:642724. doi: 10.3389/fcell.2021.642724 (PMC8027330; doi:10.3389/fcell.2021.642724)

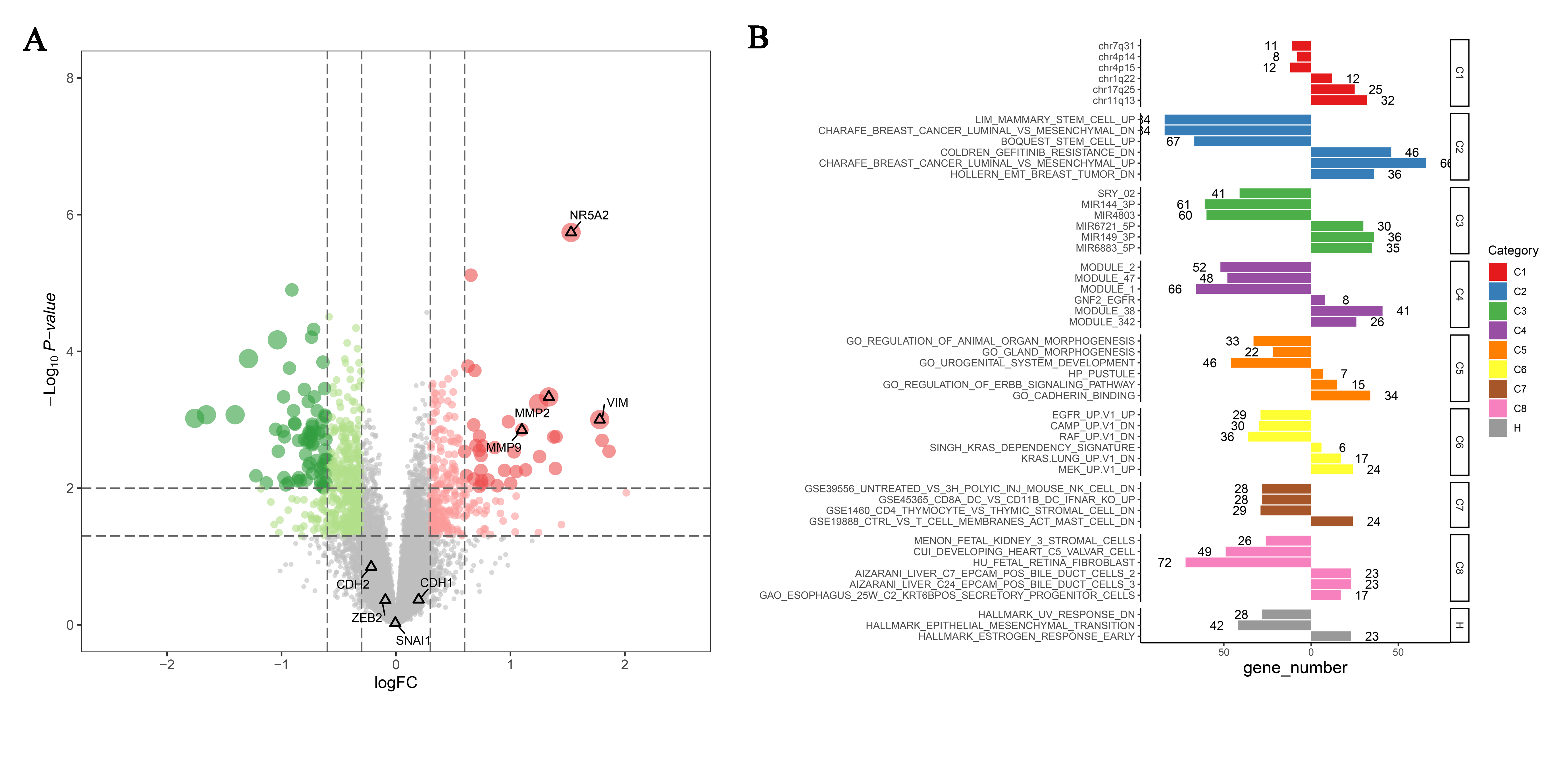

Supplement: Supplementary Figure 1 — Volcano plot for DEGs between 19 cervical cancer patients with positive lymph nodes (N+) and 20 patients with negative (N0) (A). Bar plot for gene sets over-representation analysis of DEGs and significant hallmark pathways (B). [file Image_1.TIF]
